# Evaluation of *Mallotus oppositifolius* Methanol Leaf Extract on the Glycaemia and Lipid Peroxidation in Alloxan-Induced Diabetic Rats: A Preliminary Study

**DOI:** 10.1155/2013/527205

**Published:** 2013-10-10

**Authors:** C. O. Nwaehujor, I. I. Ezeigbo, F. C. Nwinyi

**Affiliations:** ^1^Department of Biochemistry, Faculty of Basic Medical Sciences, University of Calabar, Calabar, Cross River State, Nigeria; ^2^Department of Veterinary Physiology and Pharmacology, Faculty of Veterinary Medicine, University of Nigeria, Nsukka, Enugu State, Nigeria; ^3^Department of Veterinary Physiology, Biochemistry and Pharmacology, College of Veterinary Medicine, Michael Okpara University of Agriculture, Umudike, Abia State, Nigeria; ^4^The Royal Veterinary College, University of London, London, UK; ^5^Department of Veterinary Pharmacology and Toxicology, Faculty of Veterinary Medicine, University of Abuja, Abuja, Nigeria

## Abstract

*Objective. Mallotus oppositifolius* (Geiseler) Müll. Arg. (Euphorbiaceae) is folklorically used to “treat” diabetic conditions in some parts of Nigeria therefore the study, to investigate the extract of the leaves for activities on hyperglycaemia, lipid peroxidation, and increased cholesterol levels *in vivo *in alloxan diabetic rats as well as its potential antioxidant activity *in vitro*. *Methods*. Albino rats (240–280 g) were given an injection of 120 mg/kg body weight, i.p. of alloxan monohydrate. After 8 days, diabetic animals with elevated fasting blood glucose levels (>9 mmol/L) were considered and selected for the study. *Results*. Oral treatment with the extract administered every 12 h by gavage at doses of 100, 200, and 400 mg/kg of the extract to the test rats, for 14 days, resulted in a significant dose-dependent decrease in blood glucose levels from 12.82 ± 1.02 mmol/dL to 4.92 ± 2.01 mmol/dL at the highest dose of 400 mg/kg compared to the control drug and glibenclamide as well as attendant significant decline in diabetic rats employed in the study. *Conclusion*. The extract also showed *in vitro *concentration-dependent antioxidant activity following the 1,1-diphenyl-2-picryl-hydrazyl (DPPH) and ferric reducing assays. Findings further suggest the presence of active antidiabetic and antioxidant principles in *M. oppositifolius *leaves.

## 1. Introduction

Diabetes mellitus (DM) is a multifactorial syndrome resulting from a variable interaction of hereditary and environmental factors. It is characterized by inapt hyperglycaemia caused by damaged *β*-cells of the pancreas or resistance to the action of insulin at the cellular level, or possibly a combination of both factors [1, 2]. DM affects more than 200 million people worldwide and is projected to be one of the world's major killers in the next 25 years [3], Nigeria with about 160 million people has the highest number of people with diabetes (approximately 1,338 × 10^3^), thus, the highest diabetes prevalence in the African Region (19.1%), as well as the highest number of people with impaired glucose tolerance with an estimate of about 3.85 million people. 

With on-going researches in diabetes and oral hypoglycaemic agents (OHA), the management of DM is still a global problem. One important area in diabetes management receiving particular attention today is that of herbal hypoglycaemic agents. Several factors such as dyslipidemia or hyperlipidemia which are involved in the microvascular and macrovascular complications as well as hyperglycemia are associated with the diabetes disease which is the major cause of morbidity and death [4]. There are also lipoprotein abnormalities with diabetes mellitus [5]. High blood glucose levels not only increase the production of reactive oxygen species (ROS) but also affect antioxidant activities of ROS scavenging enzymes [6, 7].

*Mallotus oppositifolius* (Geiseler) Müll. Arg. (Euphorbiaceae), locally known as “Ukpo,” is an edible plant to the South-Eastern part of Nigeria. The seed is a special soup thickener in the region. The leaves have ingredients of common antidysentery, antimalarial, and anti-inflammatory remedies [8]. Preliminary phytochemical screening of *M. oppositifolius* (MP) revealed the presence of secondary metabolites such as alkaloids, phenols, flavonoids, anthraquinones, and cardenolides [8, 9]. Iwu [10] reported five hydrolysable tannins and cytotoxic phloroglucinol from the bark of another species *Mallotus japonicus. *MP is also claimed to have antidiabetic activity by local healers in the South-East area of Nigeria. The present study was carried out to evaluate the antihyperglycemic, antioxidant, cholesterol lowering, and lipid peroxidation properties of MP in the rat model. 

## 2. Materials and Methods

### 2.1. Plant Collection and Identification

Fresh *M. oppositifolius* leaves (MP) were obtained in May 2010 from the demonstration Botanical garden of the University of Nigeria, Nsukka, and authenticated by Mr. A. Ozioko, a Taxonomist with the Biodiversity Development Centre Program (BDCP), Nsukka. Voucher samples were deposited in the herbarium of the Department of Veterinary Physiology and Pharmacology, University of Nigeria, Nsukka, where the research was carried out for reference (UN/VPP-08216). The leaves were air dried on the bench, pulverized into coarse powder, and kept in polythene bags at room temperature (23–25°C), ready for extraction. 

### 2.2. Plant Extraction

The dried pulverized leaves (1500 g) were extracted with 80% methanol in water for 48 h with intermittent shaking every 2 h. The mixtures were filtered with Whatman number 1 filter paper. The filtrates were concentrated using a rotary evaporator at 38–40°C. The water was removed using a vacuum lyophilizer, yielding 35.1 g of brownish-green powder. The powder was resuspended in distilled water before use.

### 2.3. Animals

Male albino Wistar rats (240–280 g) procured from the Animal Farm of the Faculty of Veterinary Medicine, University of Nigeria, Nsukka, were used for the study. They were kept under the following conditions temperature (25–30°C), relative humidity (40–60%) and 12 h lighting cycle, and were fed *ad libitum *with standardized rat cubes (Argo). The rats were fasted overnight before experimentation but were allowed free access to water. Ethical guidelines in animal handling and use were strictly adhered to in the execution of the study.

Diabetes was induced by a single intraperitoneal injection of 120 mg/kg body weight of alloxan monohydrate (Sigma, St. Louis, MO, USA) freshly dissolved in distilled water [11]. After 8 days, animals with fasting blood glucose level above 9 mmol/L were considered diabetic and used for the study.

Thirty mature male albino rats were randomly divided into 5 groups (Table 1). Treatment with the extract was administered every 12 h by gavage at doses of 100, 200, and 400 mg/kg of the extract to the test rats for a total of 14 days. The control drug, glibenclamide (2 mg/kg) was administered the same way for the same duration to rats in the control group.

After 14 days, being the end of the treatment period, the fasting blood glucose level of each rat was measured at 0, 1, 6, and 12 h by a snip-cut at the tip of the tail under mild anaesthesia with the aid of a glucometer (AccuChek Advantage II) as previously described [2, 12, 13]. The rats were then anaesthetized by halothane inhalation. Blood was collected from heart using disposable hypodermic syringes and transferred into EDTA tubes immediately. The blood was then centrifuged at 4000 g for 10 min to remove blood cells and recover plasma. Each rat's liver was surgically removed and washed immediately with cold-saline and stored in a freezer set at –4°C.

### 2.4. Biochemical Assays

Two (2) gram weight of liver tissues were homogenised with 20 milliliters of phosphate buffered saline (PBS), using Sonic Dismembrator (Fisher Scientific, Indiana, PA, USA). The homogenised liver tissues were used to measure lipid peroxidation using malondialdehyde (MDA) levels as markers [14]. Total cholesterol concentrations were evaluated by the methods described by Allain et al. [15] using assay kits purchased from Quimica Clinica Aplicada, S.A., Spain.

### 2.5. *In Vitro* Free Radical Scavenging Assay

The total antioxidant activity of the *Mallotus oppositifolius* (MP) extract was estimated by the ferric reducing antioxidant power (FRAP) assay [16] and the 1,1-diphenyl-2-picryl-hydrazyl (DPPH) photometric assay [17]. The procedures were carried out in triplicates and the mean used in computations.

### 2.6. Statistical Analysis

All data were analysed using SPSS version 15 (SPSS Inc., Chicago, IL) and descriptive data were expressed as mean ± SEM. Differences between the groups were separated by post hoc LSD. Using one-way ANOVA, values were considered significant at *P* ≤ 0.05.

## 3. Results

### 3.1. Plant Extraction

The powder of the 80% methanol in water extract of *Mallotus oppositifolius* was brownish-green in colour. The yield of the extract was 2.34% w/w.

### 3.2. Blood Glucose Levels

The extract decreased the blood glucose levels of experimental rats significantly (*P* ≤ 0.05), in a dose-dependent manner (Table 1). Experimental studies reveal that orally administered extracts of MP at a dose of 200 mg/kg produced significant fasting blood glucose lowering activity in 12 h compared to the control. An increase in the dose of the extract (400 mg/kg) showed an even more observable antihyperglycaemic activity in alloxanized rats. This activity at 400 mg/kg dose was very comparable to that of the reference standard, glibenclamide over the 12 h period. The variations observed in the blood glucose levels of test rats at the 0 h may be attributable to idiosyncrasies usually observed with the use of live animals in biological experiments.

### 3.3. Lipid Peroxidation Test

The study indicated a concentration dependent increase in hepatic lipid peroxidation marker-malondialdehyde (MDA) levels.  Table 2 shows the effect of the extract on the liver MDA levels of diabetic rats. The 200 mg/kg dose had values statistically significant and comparable to the control drug, glibenclamide. Rats administered the highest dose of 400 mg/kg of the extract had higher MDA levels when compared with the control, although the increase was not significant (*P* > 0.05) (Table 2).

### 3.4. Total Cholesterol Levels

**Figure 1:**
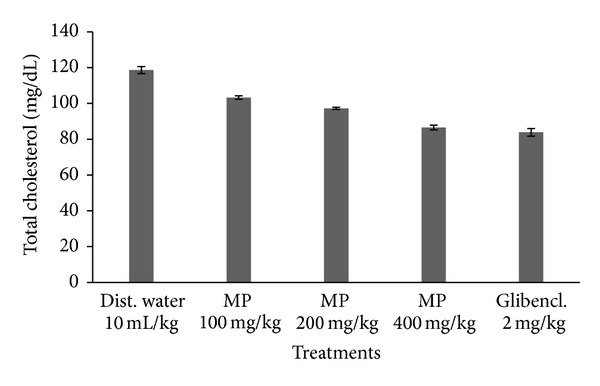
Total cholesterol levels in diabetic rats treated with various doses of MP extracts.

The total cholesterol levels of experimental animals treated with the methanol extract of MP leaves is presented in Figure 1. The results show that the levels decreased in a dose-dependent manner. The variations in the cholesterol values of the rats compared with the control groups were not significant (*P* > 0.05).

### 3.5. Free Radical Scavenging Activities

**Figure 2:**
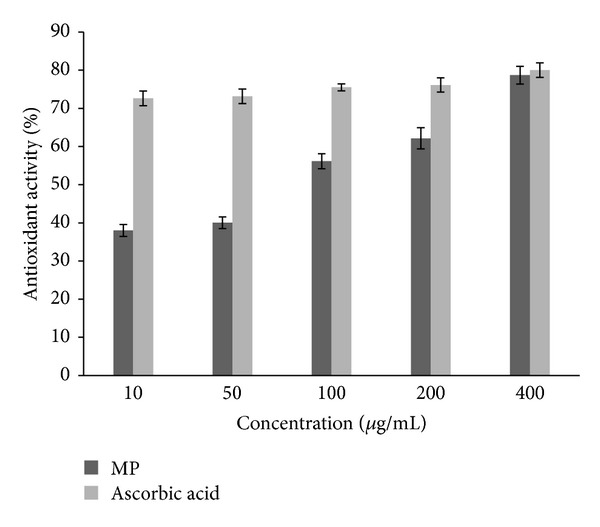
Percent antioxidant activity of MP using DPPH.

MP demonstrated appreciable antioxidant activity with DPPH radical scavenging method. At the lowest concentration (10 *μ*g/mL), the extract exhibited a mean antioxidant activity of 38% while ascorbic acid produced 72.6% (Figure 2). In the same manner, the extract elicited 56.1% antioxidant activity in contrast to 75.5% with ascorbic acid at 1000 *μ*g/mL. However, at the highest concentration (400 *μ*g/mL), the extract had 78.7% antioxidant activity compared to 80.0% with ascorbic acid at the same concentration.

**Figure 3:**
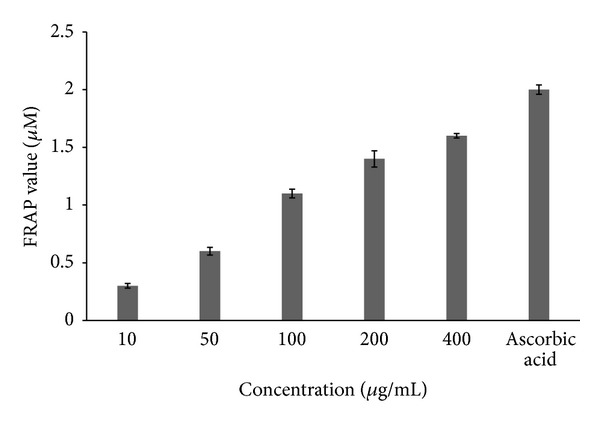
*In vitro* antioxidant activity of* M. oppositifolius* extract using the FRAP assay.

The ferric reducing ability of plasma, a measure of the antioxidant ability, showed that MP produced a dose dependent antioxidant effect. At 10 *μ*g/mL, the mean antioxidant power (FRAP) value was 0.3 *μ*M. The value however increased significantly between 100 *μ*g/mL and 400 *μ*g/mL from 1.1 *μ*M to 1.6 *μ*M, respectively (Figure 3). The frap value of the extract at the highest concentration employed in this study was still below the standard which is 1000 *μ*g/mL ascorbic acid (2.0 *μ*M). All free radical scavenging assays were carried out in triplicates and the results shown in the figures represent a mean of the values obtained.

## 4. Discussion

The diabetes disease is snowballing very fast and huge amounts of resources are spent in almost all countries to combat this multifactorial metabolic disease.

Alloxan, from the literature, has been shown to cause hyperglycaemia in laboratory animals due to increased oxidative stress by elaborated free radicals [12], thus a model for type 1 diabetes studies. Medicinal plants are effective in controlling plasma glucose levels with minimal side effects and are commonly used in under-developing and developing countries like Nigeria as alternative therapy. It has also gained popularity for decades in the treatment of diabetes mellitus [3, 18]; however, majority of the remedies have not been precisely evaluated. Therefore we selected and evaluated the methanol leaf extract of MP for its claimed antidiabetic potentials considering also, its effect on total cholesterol and biomarkers of oxidative stress *in vivo *and* in vitro*, respectively.

The results showed that MP significantly reduced the blood glucose and was found to possess significant antioxidant potentials *in vitro* both in the DPPH and the ferric reducing assay. Diabetes which is associated with impaired glucose metabolism leads to oxidative stress which with an attendant protein glycation will produce free radicals [19]. The lipid peroxidation was evaluated by measuring the raised hepatic MDA levels which lower doses (100 and 200 mg/kg) showed some significant reduction in. Further chronic toxicological evaluations on hepatocytes and other cell lines of organs in the mammalian body may be a step in the right direction.

Earlier phytochemical studies on the MP indicates the presence of large amounts of bitter tasting secondary metabolites known as alkaloids, as well as some phenolics, flavonoids and quinines in the leaves than in the roots [9, 10]. Reviews on bioactive components in plants express that desirable biological and therapeutic activities observed with plants predominantly range between the alkaloid and flavonoid components [20, 21].

Reactive oxygen species (ROS) have been implicated in many pathologies such as heart diseases, cancers, arteriosclerosis, diabetes mellitus, and arthritis [22]. Phenol compounds such as flavonoids isolated from plants scavenge for hydroxyl radicals, superoxide anion radical, and lipid peroxyl radicals unearths many of the flavonoid health promoting functions, which are important for managing diseases associated with oxidative damage such as diabetes mellitus [23]. Flavonoids in human diet may reduce the risk of various cancers as well as prevent menopausal symptoms [24]. Therefore, the antioxidant effect and the antidiabetic activities of MP are suspected to be due to its flavonoid component as observed both *in vitro* and *in vivo, *respectively.

Alkaloids of plant origin such as Solanine from *S. gilo *fruit have been linked with the improvement in the symptoms of diabetes mellitus; also those of *Momordica charantia* have been reported to improve the diabetes condition [25, 26]. Hence, it is also possible that the alkaloids present in MP may be responsible for the observed hypoglycaemic potential and associated effects on cholesterol levels in test rats.

Furthermore, increased glycogenesis, glycolysis, decreased glycogenolysis, and reduced carbohydrate breakdown as well as potential reduction in glucose absorption are further proposed as possible mechanisms of antidiabetes activity with MP.

Also, close correlations between cholesterol levels in blood and the diabetes have been previously reported [27, 28]. Elevated cholesterol (TC) and low-density lipoproteins (LDL-C) levels in the blood of diabetics are considered the primary cause of coronary heart disease (CHD) [29–31]. The reduction of TC and LDL-C by drugs and diet could reduce CHD risk [22, 32]. The reduction of cholesterol levels observed in the present study coinciding with earlier reports unearths potential cardioprotective effects also of *M. oppositifolius*. 

The findings with MP extract in this study confirm its folkloric uses in the management of diabetes, thus making *Mallotus oppositifolius* a probable plant for drug discoveries.

## 5. Conclusion

Bioactive components of the leaf extracts of *M. oppositifolius* may suggest multiple mechanisms of action in the control of blood and cholesterol levels.

Further studies will be needed to isolate and purify the bioactive compound(s) in the extract for bioassay-guided experiments in a view to pin-point its mechanisms of antidiabetic action. MP poses as a potential for ground-breaking diabetes study.

## Figures and Tables

**Table 1:** The effect of MP extract on the fasting blood glucose levels of test rats.

Group	Treatment	No. of animals	Weight	Blood glucose levels in mmol/L			
				Sampling time in hours			
				0	1	6	12
I	Negative control (dist. water), that is, Diabetic rats	6	220.44 ± 1.55	15.28 ± 1.89 (0.231)	16.26 ± 1.46 (0.817)	17.18 ± 1.98 (0.280)	17.72 ± 2.14 (0.121)
II	Diabetic rats treated with MP 100 mg/kg	6	232.54 ± 0.83	11.48 ± 0.69 (0.342)	11.06 ± 0.77 (0.108)	10.14 ± 0.62* (0.021)	9.12 ± 1.61 (0.075)
III	Diabetic rats treated with MP 200 mg/kg	6	233.92 ± 1.85	11.22 ± 0.37 (0.583)	9.04 ± 0.52* (0.005)	7.36 ± 0.35* (0.014)	7.02 ± 1.10 (0.770)
IV	Diabetic rats treated with MP 400 mg/kg	6	220.46 ± 1.15	12.82 ± 1.02 (0.021)	9.88 ± 0.58** (0.0001)	5.06 ± 0.65* (0.013)	4.92 ± 2.01* (0.04)
V	Positive control-diabetic rats treated with glibenclamide (2 mg/kg)	6	221.42 ± 2.68	11.38 ± 1.13 (0.108)	7.14 ± 0.22* (0.034)	4.72 ± 0.63 (0.280)	4.01 ± 1.03* (0.033)

Data are expressed as mean ± SEM. *P values ( ) < 0.05 **P values ≤ 0.001 compared to the control groups were considered significant.

**Table 2:** Malondialdehyde (MDA) levels in the liver of diabetic rats treated with different doses of MP extract.

Groups	Treatments	No. of animals	MDA levels (g/g tissue) mean ± SEM
I	Dist. Water	6	0.866 ± 0.02 (0.159)
II	MP 100 mg/kg	6	0.154 ± 0.01* (0.031)
III	MP 200 mg/kg	6	0.173 ± 0.03** (0.001)
IV	MP 400 mg/kg	6	1.401 ± 0.04 (0.121)
V	Glib. (2 mg/kg)	6	0.187 ± 0.10* (0.017)

Data are expressed as mean ± SEM. *P values () < 0.05, **P values < 0.001 compared to the control groups were considered significant.

## References

[1] Vinuthan MK, Kumara G, Narayanaswamya M, Veena T (2007). Lipid lowering effect of aqueous leaves extract of *Murraya koenigii* (curry leaf) on alloxan-induced male diabetic rats. *Pharmacognosy Magazine*.

[2] Ezeigbo II (2010). Antidiabetic potential of methanolic leaf extracts of *Icacina trichantha* in alloxan-induced diabetic mice. *International Journal of Diabetes in Developing Countries*.

[3] Jayakumar RV (2010). Herbal medicines for type-2 diabetes. *International Journal of Diabetes in Developing Countries*.

[4] Taskinen MR (1987). Lipoprotein lipase in diabetes. *Diabetes/Metabolism Reviews*.

[5] Scoppola A, Montecchi FR, Menzinger G, Lala A (2001). Urinary mevalonate excretion rate in type 2 diabetes: role of metabolic control. *Atherosclerosis*.

[6] Uchimura K, Nagasaka A, Hayashi R (1999). Changes in superoxide dismutase activities and concentrations and myeloperoxidase activities in leukocytes from patients with diabetes mellitus. *Journal of Diabetes and Its Complications*.

[7] Ugochukwu NH, Babady NE, Cobourne M, Gasset SR (2003). The effect of *Gongronema latifolium* extracts on serum lipid profile and oxidative stress in hepatocytes of diabetic rats. *Journal of Biosciences*.

[8] Kabran FA, Maciuk A, Okpekon TA (2012). Phytochemical and biological analysis of *M. oppositifolius* (Euphorbiaceae). *Planta Medica*.

[9] Farombi EO, Ogundipe OO, Moody JO (2001). Antioxidant and anti-inflammatory activities of *Mallotus oppositifolium* in model systems. *African Journal of Medicine and Medical Sciences*.

[10] Iwu MM (1993). *Handbook of African Medicinal Plants*.

[11] Anthony MU, Adebimpe AO (2009). Hypoglycemic potential of the young leave methanolic extract of *Mangifera indica* in alloxan induced diabetic rat. *Pakistan Journal of Nutrition*.

[12] Ezeigbo II (2011). The antidiabetic potentials of the methanolic seed extract of *Buchhlozia coriacea*. *Annals of Medical and Health Sciences Research*.

[13] Malviya N, Jain S, Malviya S (2010). Antidiabetic potential of medicinal plants. *Acta Poloniae Pharmaceutica—Drug Research*.

[14] Sunderman FW, Marzouk A, Hopfer SM, Hopfer SM, Zaharia O, Reid MC (1985). Increased lipid peroxidation in tissues of nickel chloride-treated rats. *Annals of Clinical and Laboratory Science*.

[15] Allain CC, Poon LS, Chan CSG (1974). Enzymatic determination of total serum cholesterol. *Clinical Chemistry*.

[16] Benzie FF, Strain JJ (1998). Ferric reducing/antioxidant power assay: direct measure of total antioxidant activity of biological fluids and modified version for simultaneous measurement of total antioxidant power and ascorbic acid concentration. *Methods in Enzymology*.

[17] Mensor LL, Menezes FS, Leitão GG (2001). Screening of Brazilian plant extracts for antioxidant activity by the use of DPPH free radical method. *Phytotherapy Research*.

[18] Schmelzer GH, Achigan-Dako EG, Bosch CH (2010). *Medicinal Plants of Tropical Africa*.

[19] Wolff SP, Jiang ZY, Hunt JV (1991). Protein glycation and oxidative stress in diabetes mellitus and ageing. *Free Radical Biology and Medicine*.

[20] Okigbo RN, Anuagasi CL, Amadi JE (2009). Advances in selected medicinal and aromatic plants indigenous to Africa. *Journal of Medicinal Plant Research*.

[21] Middleton E, Kandaswami C, Theoharides TC (2000). The effects of plant flavonoids on mammalian cells: implications for inflammation, heart disease, and cancer. *Pharmacological Reviews*.

[22] Gutstein DE, Fuster V (1999). Pathophysiology and clinical significance of atherosclerotic plaque rupture. *Cardiovascular Research*.

[23] Sofidiya MO, Odukoya OA, Familoni OB, Inya-Agha SI (2006). Free radical scavenging activity of some Nigerian medicinal plant extracts. *Pakistan Journal of Biological Sciences*.

[24] Hodek P, Trefil P, Stiborová M (2002). Flavonoids-potent and versatile biologically active compounds interacting with cytochromes P450. *Chemico-Biological Interactions*.

[25] Krawinkel MB, Keding GB (2006). Bitter gourd (*Momordica charantia*): a dietary approach to hyperglycemia. *Nutrition Reviews*.

[26] Grover JK, Yadav S, Vats V (2002). Medicinal plants of India with anti-diabetic potential. *Journal of Ethnopharmacology*.

[27] Azad K, Parkin JM, Court S, Laker MF, Alberti KGMM (1994). Circulating lipids and glycaemic control in insulin dependent diabetic children. *Archives of Disease in Childhood*.

[28] Diwadkar VA, Anderson JW, Bridges SR, Gowri MS, Oelgten PR (1999). Postprandial low-density lipoproteins in type 2 diabetes are oxidized more extensively than fasting diabetes and control samples. *Proceedings of the Society for Experimental Biology and Medicine*.

[29] Rodrigues B, McNeil JH (1986). Cardiac function in spontaneously hypertensive diabetic rats. *The American Journal of Physiology—Heart and Circulatory Physiology*.

[30] Genest JJ, McNamara JR, Salem DN, Schaefer EJ (1991). Prevalence of risk factors in men with premature coronary artery disease. *The American Journal of Cardiology*.

[31] Sharma MD, Farmer JA, Garber A (2011). Type 2 diabetes and cardiovascular risk factors. *Current Medical Research and Opinion*.

[32] Levine GN, Keaney JF, Vita JA (1995). Cholesterol reduction in cardiovascular disease—clinical benefits and possible mechanisms. *The New England Journal of Medicine*.

